# Global research status and hotspots of radiotherapy for prostate cancer: a bibliometric analysis based on Web of Science from 2010-2022

**DOI:** 10.3389/fonc.2023.1135052

**Published:** 2023-08-11

**Authors:** Xiaodu Xie, Peng Zhang, Chongjun Ran, Lumiao Liu, Jian Hu, Pan Lei, Peihe Liang

**Affiliations:** ^1^ Department of Urology, The Second Affiliated Hospital of Chongqing Medical University, Chongqing, ;China; ^2^ Department of Anesthesiology, The Second Affiliated Hospital of Chongqing Medical University, Chongqing, China

**Keywords:** radiotherapy, prostate cancer, bibliometric, CiteSpace, VOSviewer, hotspots

## Abstract

**Background:**

Radiotherapy (RT) is one of the important treatments for various cancer types and its application to prostate cancer (PCa) has also gradually gained increasing attention. However, there is a lack of comprehensive and objective studies on the overall status of research on RT for PCa. This article aims to summarize and quantify the dynamic trends of RT in PCa by using bibliometrics.

**Methods:**

Studies on RT for PCa were screened from the Web of Science Core Collection (WoSCC) database between 1 January 2010 and 21 November 2022 to collate and quantify information characteristics by analyzing parameters including annual publications, countries/regions, institutions and authors with the aid of the bibliometric software CiteSpace and VOSviewer. In addition, research trends and hotspots were explored by analyzing keywords and co-cited references.

**Results:**

A total of 21338 documents were retrieved. The United States of America (USA) ranked first and maintained the leading position among all countries in the number of publications (8489) and total citations (266342). The University of Toronto was the most active institution in total publications (n=587). Paul L Nguyen enjoyed the most publications (n=179), and Michael J Zelefsky enjoyed the most co-citations (n=3376). *INTERNATIONAL JOURNAL OF RADIATION ONCOLOGY BIOLOGY PHYSICS* published the most papers (n=1026), and was the most frequently co-cited journal (n=78550). The largest and closest cluster in the reference cluster analysis was “oligorecurrent prostate cancer”. The timeline view of keywords reveals that cluster “biochemical recurrence(BCR)” is ongoing. Moreover, keywords burstness analysis showed that “radiation dosimetry”, “dose rate brachytherapy(BT)”, “salvage radiotherapy”, “stereotactic body radiotherapy(SBRT)”, “guideline”, and “multicenter” were the terms with great bursts in the past a few years.

**Conclusion:**

The application of RT targeting oligometastatic prostate cancer(OMPC) has garnered considerable attention among researchers. SBRT and BT have become hot topics in the field. Additionally, the BCR of PCa has long been a critical issue requiring extensive research and resolution, and salvage radiotherapy has currently emerged as a closely related research focus. Related large-scale multicenter studies have been conducted over the past few years, providing valuable insights. More high-quality research is expected to be employed to guide clinical decision-making.

## 1 Introduction

Prostate cancer (PCa) is an epithelial malignant tumor occurring in the male prostate gland and is the most common malignant tumor of the male genitourinary system. According to data released by the National Comprehensive Cancer Network (NCCN) in 2018, PCa has surpassed lung cancer as the most common malignancy in men and ranks the second leading cause of cancer-related death in men worldwide (1). Radical prostatectomy (RP), radiotherapy (RT) and endocrine therapy remain the principal treatments for PCa at present. RT plays an irreplaceable role in radical RT, postoperative adjuvant or salvage RT and palliative care due to minimal trauma, high safety and the reliable curative effect. RT for PCa, eighter used alone or in combination with other treatments, is a widely accepted. The use of RT as adjuvant treatment after radical prostatectomy has proved to improve progression-free survival (PFS) and reduce the incidence of associated adverse events (2, 3). With the deeper understanding of the radiobiological behavior of PCa and the advent of new techniques, more advances have been made in RT of PCa patients.

In recent years, bibliometrics has emerged as a crucial academic field, focusing on quantifying and evaluating the quantitative attributes, developmental trends, and scholarly impact of scientific literature. While a quantitative overview can be drawn on many methods such as traditional reviews, meta-analysis, and evidence maps, only bibliometrics allows for a qualitative and quantitative analysis of data characteristics such as countries, institutions, authors, and journals, as well as an assessment of trends and profiles of research topics (4, 5). RT, as a pivotal modality in the management of PCa, encompasses a diverse array of treatment modalities, including external beam radiation therapy (EBRT), brachytherapy (BT), and proton therapy, among others. Several research teams have embarked on bibliometric investigations about the application of EBRT in PCa, uncovering salient trends and focal points of interest in this domain (6). However, it is noteworthy that there is currently a lack of sufficient bibliometric research specifically addressing the entire domain of RT applications in PCa. Conducting a bibliometric analysis encompassing the entire domain of RT to explore its focal points and advancements in PCa will facilitate a macroscopic comprehension of the potential strengths and challenges of RT in PCa treatment, consequently furnishing more robust scientific grounds for future clinical practices and therapeutic strategies.

Based on the above background and theoretical support, this paper aims to provide an overall picture of research on RT in PCa and address the research progress, hotspots and trends in the last decade by using two bibliometric software VOSviewer and CiteSpace, in an attempt to provide useful references for future research in this field.

## 2 Materials and methods

### 2.1 Data collection

**Figure 1:**
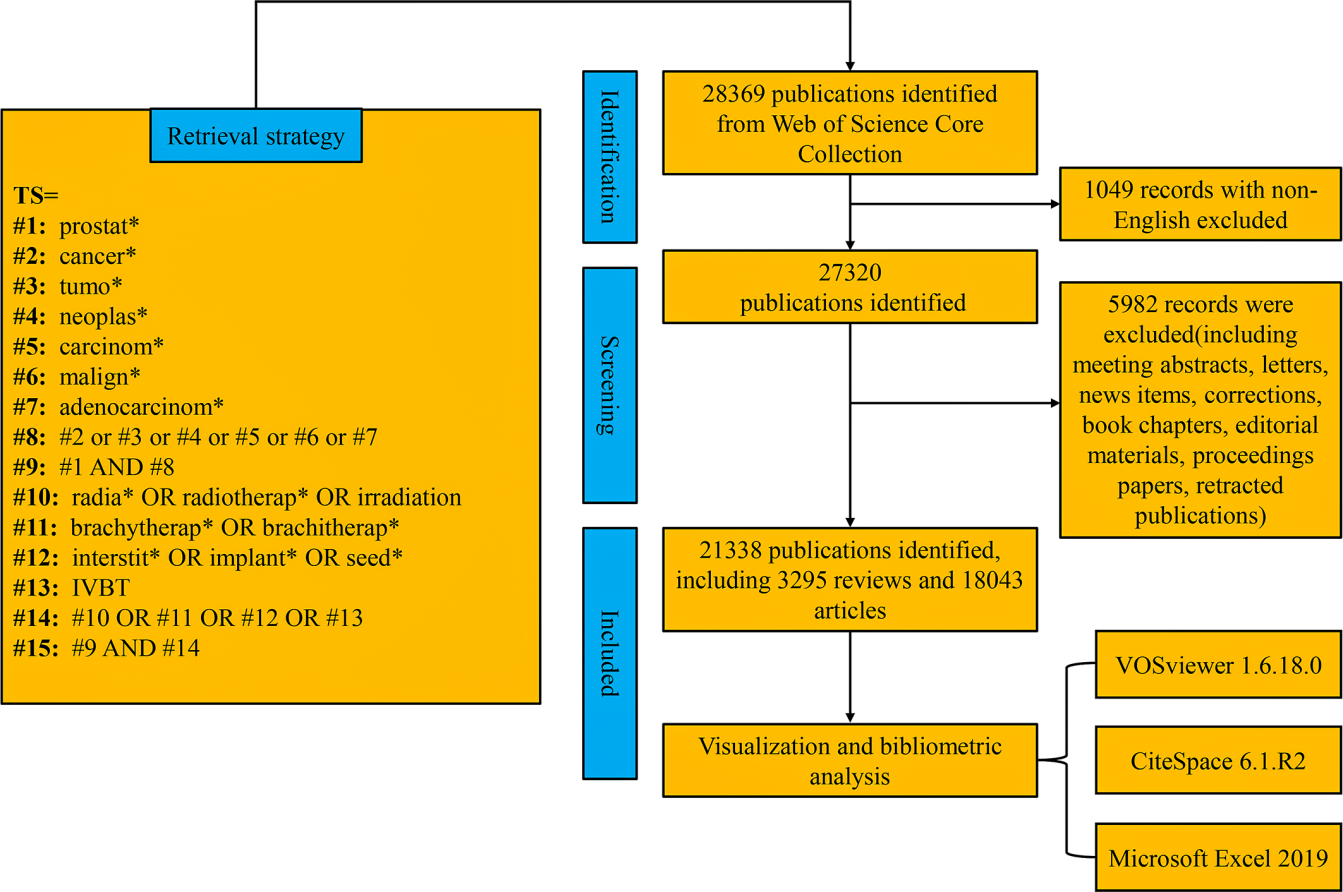
Flow chart of data screening. TS, topic search; *, truncation operator; #, connection character.

Web of Science (WoS) is an important platform for obtaining global academic information, containing databases such as Science Citation Index Expanded (SCIE), Social Science Citation Index (SSCI), and Conference Proceedings Citation Index (CPCI-S), which include more than 10,000 authoritative and high-impact international academic journals. In this study, we collected and analyzed data by searching the Science Citation Index Expanded Web of Science Core Collection (WoSCC) database. To avoid omissions caused by frequent updates of the database, document retrieval and data download were completed within one day (November 21, 2022). The search formula and process of data screening are shown in **Figure 1**, with the publication year ranging from January 1^st^, 2010 to November 21^st^, 2022. Only reviews and original articles published in English were included in this study. The search process was conducted independently by two individuals, and in case of disagreement, the final decision would be made by the third more experienced corresponding author.

### 2.2 Bibliometric analysis

Bibliometric analysis and visualization were performed by using CiteSpace 6.1.R2, VOSviewer 1.6.18.0, and Microsoft Excel 2019, knowing that CiteSpace is a Java application for identifying and displaying new trends and developments in the scientific literature developed by Professor Chen Chaomei (7), and CiteSpace software makes it possible to find out research advances and current research frontiers in a certain subject area and its corresponding knowledge base (8, 9). We deployed CiteSpace to perform the dual-map overlay of journals, cluster and burstness analysis of references, and timeline and burstness analysis of keywords. The parameters were set as follows: the minimum burst duration (1 year), time span (January 2010 to December 2022), pruning (painfinder and pruning sliced networks), and selection criteria (Top N=50). The cluster analysis was performed by the log-likelihood ratio (LLR) algorithm, and other parameters were set to default values. In addition, we further calculated the nodes with high betweenness centrality (≥0.1) in the keywords to identify the important pivots within a domain (7, 9).

VOSviewer is another professional bibliometric analysis and knowledge graph visualization software suitable for large-scale data analysis, which supports labeled views, overlay views, density views, and cluster views (10). In this study, VOSviewer software was used to map the country/region collaboration network, author collaboration network along with co-citation network, journals co-citation network, references co-citation network, and co-occurrence network of keywords. All the contents were analyzed by the fractional counting method, with the cartographic thresholds shown in the corresponding sections.

Excel software was used to collate data characteristics. The graph of the annual publication quantity in the top 10 countries/regions was created with the help of an online website (https://bibliometric.com/). In addition, Journal Citation Reports (JCR), as an authoritative multidisciplinary journal evaluation tool, is an important indicator to measure the value of scientific research. The H-index can also accurately measure an author’s academic achievement (11). We obtained the JCR division and impact factor (IF) of journals in 2021, as well as the H-index of researchers through the WoS database.

## 3 Results

### 3.1 Contributions of countries/regions and institutions to global publications

**Figure 2:**
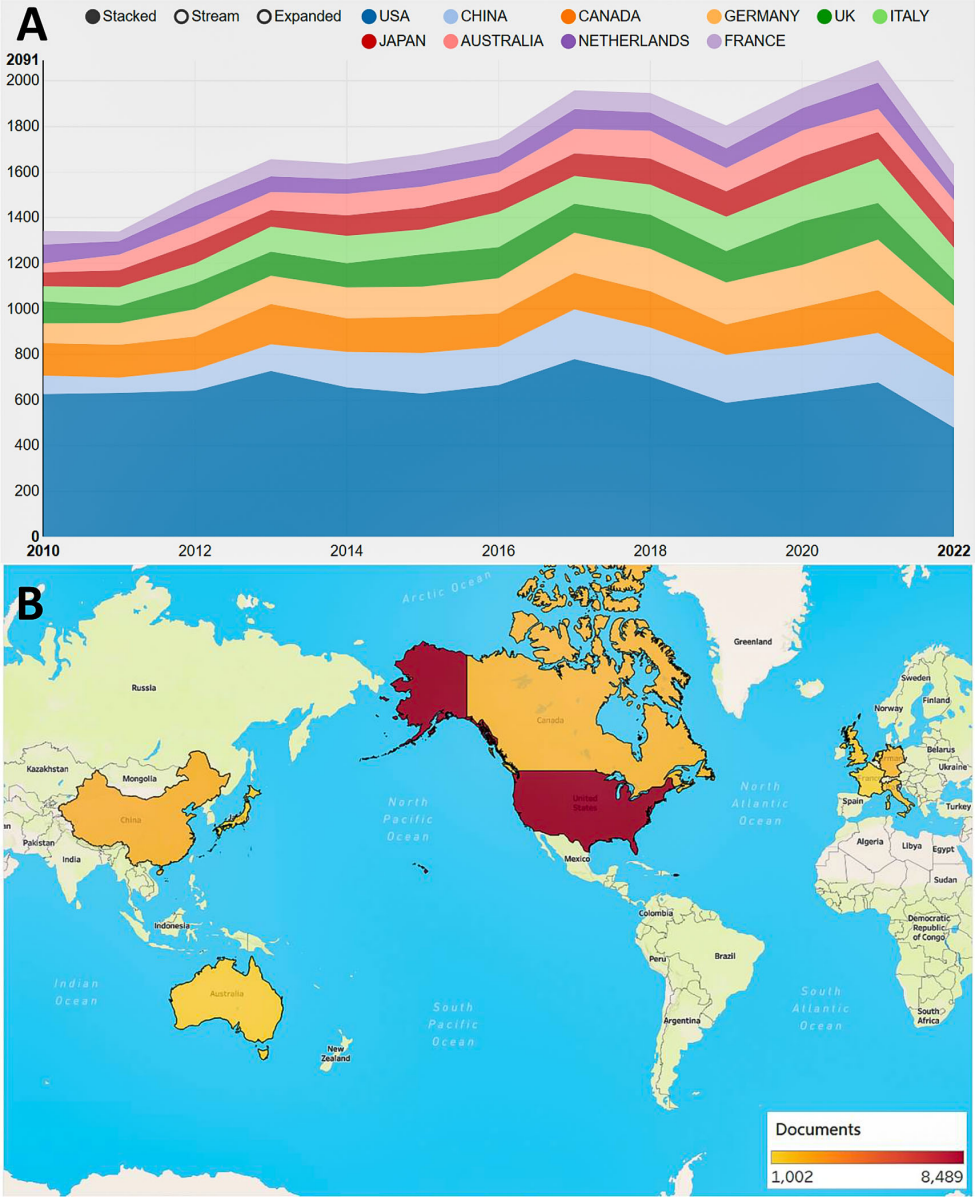
Analysis of publications in countries/regions. **(A)** Annual publication trends for the top 10 countries by the article number. **(B)** Geographical map of the top 10 countries/regions in the number of publications.

**Figure 3:**
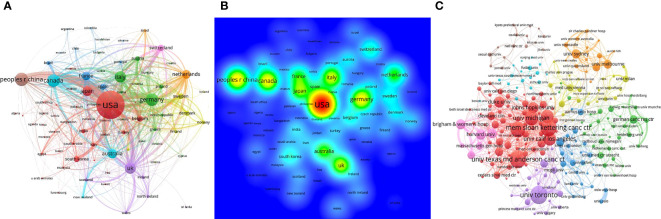
Analysis of cooperation networks in countries/regions and institutions. **(A)** National cooperation network. The size of the node represents the number of documents, and the thickness of links represents the strength of collaboration. **(B)** Density map of countries and regions. **(C)** Cooperation network visualization between institutions.

A total of 21338 papers (18043 original articles and 3295 reviews) were screened from the WoSCC database, involving 129 countries/regions and 14184 institutions (**Figure 1**). Over the past 10 years, studies related to RT in PCa have increased steadily. The United States of America (USA) took the lead in the annual publication volume (**Figure 2A**). The top 10 countries/regions and institutions by the number of publications are shown in **Table 1**. The USA enjoyed the largest number of papers (n=8489), followed by China (n=2198), Canada (n=2028), and Germany (n=1972) (**Figure 2B**). However, China only ranked eight in citations (n= 38827), with the USA taking the first place (n= 266342), and the United Kingdom (UK) the second place (n= 69001), and Canada in third place (n= 67039). As shown in the country cooperation map, the intensity of cooperation between China and the USA was the strongest, and the cooperation between the other countries was comparatively weak (**Figure 3A**). The national cooperation as a whole needed to be strengthened in future. **Figure 3B** further demonstrates the dominance of *the Occident*, showing that the USA took the lead in this field. The institutional cooperation network in **Figure 3C** shows that most of the top 10 publishers were in the USA (n=9, 90%). It is noteworthy that the University of Toronto was the institution with the largest number of papers (n=587). The contribution of Canada to the field of RT in PCa also deserves close attention.

**Table 1:** Top 10 countries/regions and institutions related to radiotherapy for prostate cancer.

Rank	Country/Regions	Count	Citations	Rank	Institution	Count	Citations
1	United States	8489	266342	1	University of Toronto, Canada	587	21029
2	China	2198	38827	2	Memorial Sloan-Kettering Cancer Center, USA	553	25803
3	Canada	2028	67039	3	The University of Texas MD Anderson Cancer Center, USA	551	20415
4	Germany	1972	64985	4	University of California - San Francisco, USA	484	17668
5	Italy	1634	49370	5	University of Michigan, USA	448	16889
6	United Kingdom	1563	69001	6	University of California, Los Angeles, USA	409	16097
7	Japan	1280	18821	7	Mayo Clinic, USA	407	15101
8	Australia	1186	36198	8	The Johns Hopkins University, USA	327	12115
9	Netherlands	1037	44013	9	Duke University, USA	327	13249
10	France	1002	40915	10	Harvard University, USA	307	19204

### 3.2 Analysis of authors

**Figure 4:**
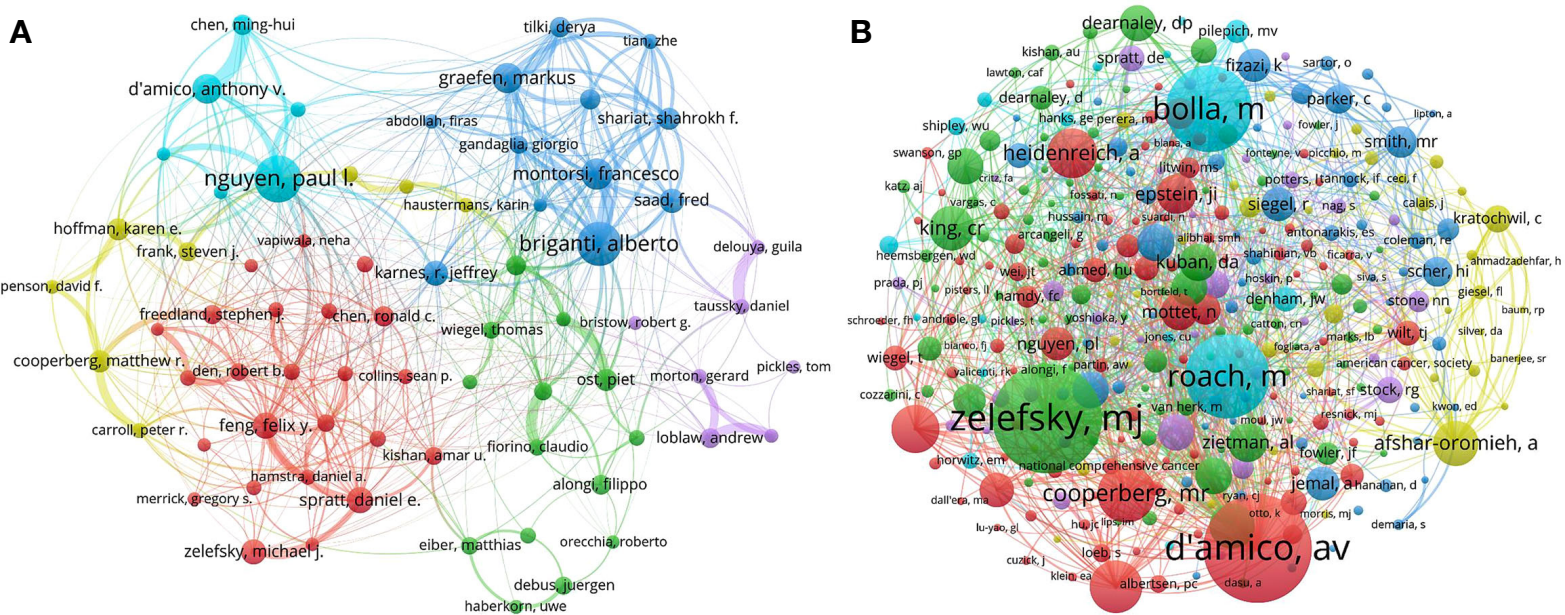
Visualization map of authors. **(A)** Author collaboration network. The size of the node indicates the number of papers, and the thickness of the links represents the intensity of the cooperation. **(B)** Author co-citation analysis.

Of the 82648 authors selected by VOSviewer, 71 authors had 50 or more publications, based on which an author collaboration network was drawn (**Figure 4A**). The six colors in the cooperation network represent different clusters. The high cooperation intensity mainly occurred in the same cluster, such as “D’amico, Anthony V.” and “Chen, Ming-Hui”, “Graefen, Markus” and “Tilki, Derya”. It is clear that “Briganti, Alberto” was at the center of the collaborative network, with a high level of collaboration with other authors. The author with the largest number of articles in the field was “Nguyen, Paul L.”(n=179), followed by “Briganti, Alberto” (n=168) and “Montorsi, Francesco”(n=121) (**Table 2**).

**Table 2:** Top 10 authors and co-cited authors related to radiotherapy for prostate cancer.

Rank	Author	Documents	H-Index	Author	Co-citations	H-Index
1	Nguyen, Paul L.	179	56	Zelefsky, Michael J.	3376	86
2	Briganti, Alberto	168	83	D'amico, Anthony V.	3347	71
3	Montorsi, Francesco	121	115	Bolla, Michel	2650	47
4	Graefen, Markus	117	85	Roach, Mack	2574	61
5	D'amico, Anthony V.	114	71	Cooperberg, Matthew R.	1731	65
6	Feng, Felix Y.	100	79	Thompson, Ian M.	1568	82
7	Spratt, Daniel E.	100	27	Heidenreich, Axel	1481	67
8	Karnes, R. Jeffrey	96	64	Stephenson, Andrew J.	1443	52
9	Zelefsky, Michael J.	96	90	Afshar-Oromieh, Ali	1349	37
10	Saad, Fred	93	86	Pollack, Alan	1344	51

In addition, co-cited authors refer to two or more authors who are simultaneously cited in one or more papers. Among the 209840 co-cited authors, 332 authors enjoyed more than 200 co-citations (**Figure 4B**). Larger nodes represent more citations. The top three authors with the most co-citations were “Zelefsky, Michael J. “(n=3376), “D’amico, Anthony V.”(n=3347), and “Bolla, Michel”(n=2650) (**Table 2**).

### 3.3 Analysis of journals

**Figure 5:**
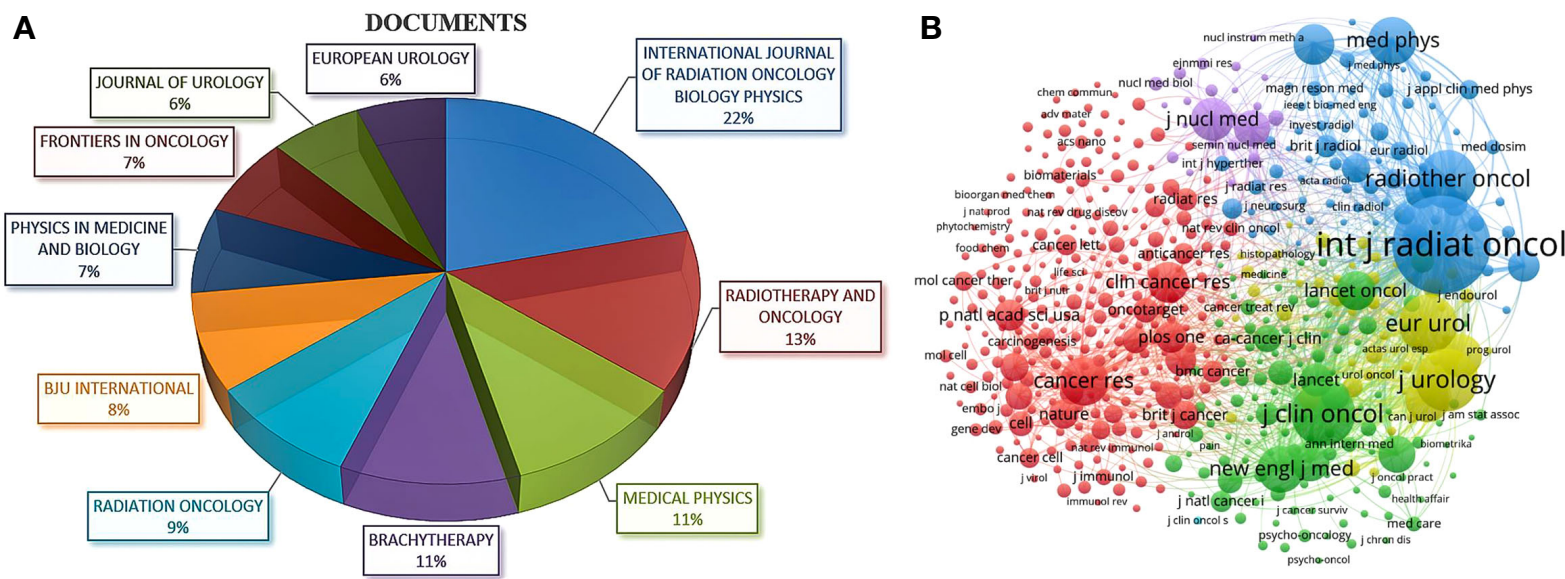
Visualization map of journals. **(A)** Pie chart of top 10 journals that published the largest number of documents. **(B)** Co-citation network of journals based on the reference sources. The size of the nodes indicates the co-citations of each journal, and the lines between the nodes represent the link strength.

A total of 2005 journals published articles on RT in PCa, of which the top 10 journals published 4447 publications, accounting for 22.4% of all papers (**Table 3**). The journal with the most publications was *INTERNATIONAL JOURNAL OF RADIATION ONCOLOGY BIOLOGY PHYSICS* (n=1026, IF=8.013), followed by *RADIOTHERAPY AND ONCOLOGY* (n=641, IF=6.901) and *MEDICAL PHYSICS* (n=515, IF=4.506) (**Figure 5A**). In addition, analysis of co-cited journals can determine the core or marginal position of a journal in a discipline. Highly co-cited journals represent their significant influence in a specific field. Of the 34902 co-cited journals, 544 journals were cited more than 200 times, with *INTERNATIONAL JOURNAL OF RADIATION ONCOLOGY BIOLOGY PHYSICS* (n=78550, IF=8.013) taking the lead, followed by *JOURNAL OF CLINICAL ONCOLOGY* (n=30749, IF=50.739) and *JOURNAL OF UROLOGY* (n=29698, IF=7.641) (**Table 3**). The corresponding co-citation network diagram is shown in **Figure 5B**, which contains five clusters.

**Table 3:** Top 10 journals and co-cited journals related to radiotherapy for prostate cancer.

Rank	Journal	Documents	JCR(2021)	IF(2021)	Co-cited journal	Citations	JCR(2021)	IF(2021)
1	INTERNATIONAL JOURNAL OF RADIATION ONCOLOGY BIOLOGY PHYSICS	1026	Q1	8.013	INTERNATIONAL JOURNAL OF RADIATION ONCOLOGY BIOLOGY PHYSICS	78550	Q1	8.013
2	RADIOTHERAPY AND ONCOLOGY	641	Q1	6.901	JOURNAL OF CLINICAL ONCOLOGY	30749	Q1	50.739
3	MEDICAL PHYSICS	515	Q2	4.506	JOURNAL OF UROLOGY	29698	Q1	7.641
4	BRACHYTHERAPY	501	Q3	2.441	EUROPEAN UROLOGY	25582	Q1	24.344
5	RADIATION ONCOLOGY	425	Q2	4.309	RADIOTHERAPY AND ONCOLOGY	25472	Q1	6.901
6	BJU INTERNATIONAL	390	Q1	5.969	CANCER RESEARCH	17051	Q1	13.312
7	PHYSICS IN MEDICINE AND BIOLOGY	348	Q2	4.174	MEDICAL PHYSICS	16903	Q2	4.506
8	FRONTIERS IN ONCOLOGY	326	Q2	5.738	NEW ENGLAND JOURNAL OF MEDICINE	15671	Q1	176.082
9	JOURNAL OF UROLOGY	305	Q1	7.641	UROLOGY	14217	Q3	2.633
10	EUROPEAN UROLOGY	300	Q1	24.344	BJU INTERNATIONAL	13346	Q1	5.969

IF, impact factor; JCR, journal citation reports; Q, quartile in category.

**Figure 6:**
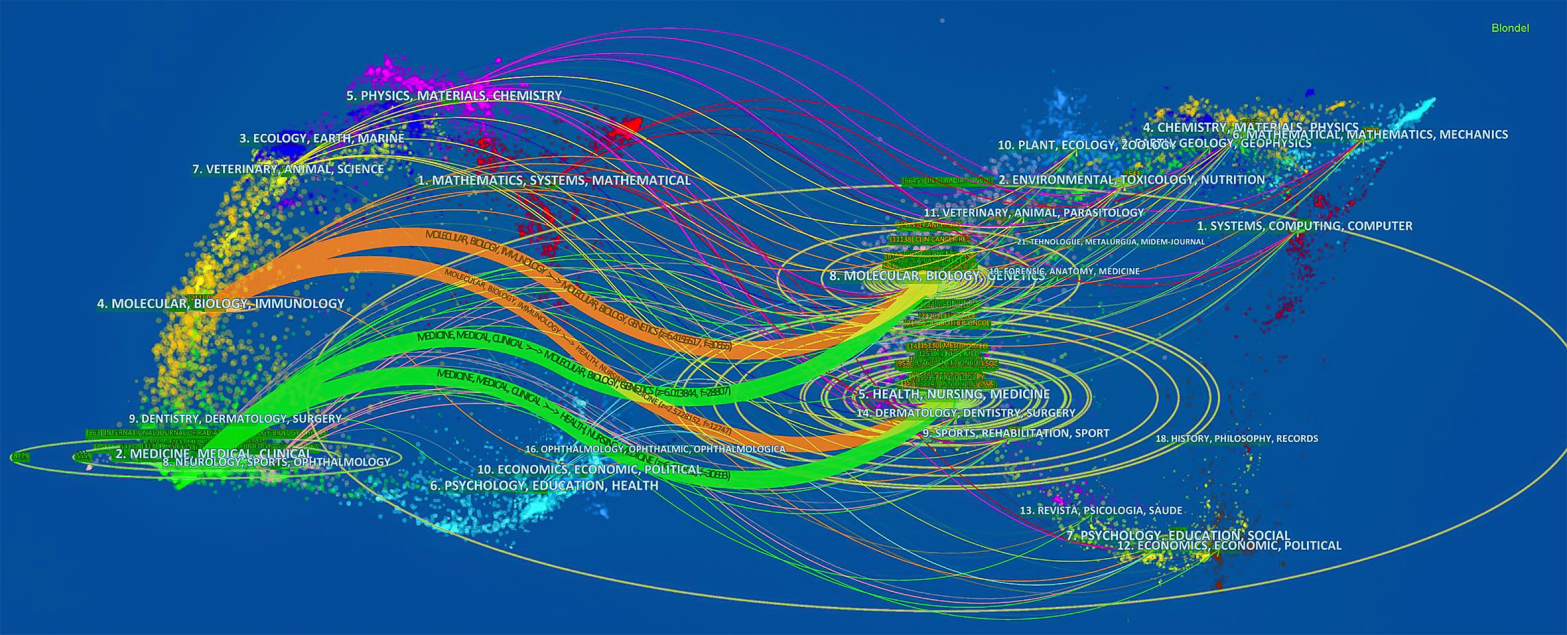
The dual-map overlay of journals on radiotherapy for prostate cancer.

Additionally, the topic distribution of academic journals is represented by conducting the dual-map overlay of journals (**Figure 6**). Citing journals are on the left and cited journals are on the right, with colored lines standing for citation relationships. It can be seen that there are mainly four paths, from Medicine/Medical/Clinical journals to Molecular/Biology/Genetics journals, Medicine/Medical/Clinical journals to Health/Nursing/Medicine journals, Molecular/Biology/Immunology journals to Molecular/Biology/Genetics journals, and Molecular/Biology/Immunology journals to Health/Nursing/Medicine journals.

### 3.4 Co-citation network of references

**Figure 7:**
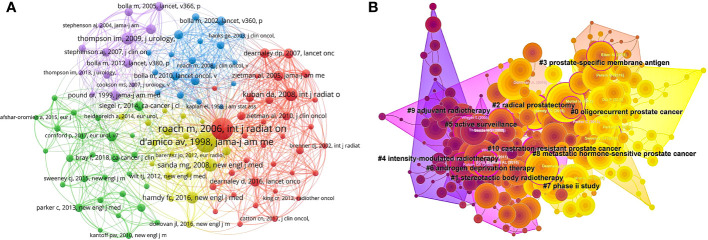
Visualization diagram of co-cited references. **(A)** Co-citation network of references. **(B)** Cluster analysis of cited references related to radiotherapy for prostate cancer. The color approaching yellow represents time getting closer.

Of the 417830 cited references, 112 were cited at least 200 times, and the corresponding co-citation network is shown in **Figure 7A**. **Table 4** presents the top 10 cited references, all of which are articles. The most cited reference was written by Mack Roach 3rd et al. in *INTERNATIONAL JOURNAL OF RADIATION ONCOLOGY BIOLOGY PHYSICS* in 2006, which is entitled *Defining biochemical failure following radiotherapy with or without hormonal therapy in men with clinically localized prostate cancer: recommendations of the RTOG-ASTRO Phoenix Consensus Conference* (n=1285). We then performed a cluster analysis of the references. The largest 11 clusters are summarized in **Figure 7B**. The clustering color tends to be yellow to indicate a more recent occurrence. The largest and closest cluster was #0(oligorecurrent prostate cancer), to which the most relevant citer was *French ccafu guidelines - update 2020-2022: prostate cancer.* These updated French guidelines highlight the need for early salvage RT in the presence of biochemical recurrence (BCR) after RP and point out that the application of RT as a localized treatment modality for PCa can improve survival in synchronous OMPC patients (12). OMPC has emerged as a prominent research focus in recent years, prompting numerous clinical trials. For instance, Ren et al. conducted the world’s first phase I/II prospective clinical trial on the “sandwich” therapy of OMPC, demonstrating the favorable tolerability of neoadjuvant radiohormonal therapy in OMPC patients (13). This breakthrough study provides a novel perspective for the treatment of advanced prostate cancer patients, presenting a new avenue for therapeutic exploration.

**Table 4:** Top 10 co-cited references related to radiotherapy for prostate cancer.

Rank	Title	Type	Year	First Author	Journals	Citations
1	Defining biochemical failure following radiotherapy with or without hormonal therapy in men with clinically localized prostate cancer: recommendations of the RTOG-ASTRO Phoenix Consensus Conference	Article	2006	Mack Roach 3rd	International journal of radiation oncology, biology, physics	1285
2	Biochemical outcome after radical prostatectomy, external beam radiation therapy, or interstitial radiation therapy for clinically localized prostate cancer	Article	1998	A V D'Amico	JAMA	1182
3	Long-term results of the M. D. Anderson randomized dose-escalation trial for prostate cancer	Article	2008	Deborah A Kuban	International journal of radiation oncology, biology, physics	686
4	Quality of life and satisfaction with outcome among prostate-cancer survivors	Article	2008	Martin G Sanda	The New England journal of medicine	670
5	Adjuvant radiotherapy for pathological T3N0M0 prostate cancer significantly reduces risk of metastases and improves survival: long-term followup of a randomized clinical trial	Article	2009	Ian M Thompson	The Journal of urology	622
6	10-Year Outcomes after Monitoring, Surgery, or Radiotherapy for Localized Prostate Cancer	Article	2016	Freddie C Hamdy	The New England journal of medicine	593
7	Natural history of progression after PSA elevation following radical prostatectomy	Article	1999	C R Pound	JAMA	503
8	Comparison of conventional-dose vs high-dose conformal radiation therapy in clinically localized adenocarcinoma of the prostate: a randomized controlled trial	Article	2005	Anthony L Zietman	JAMA	498
9	Escalated-dose versus standard-dose conformal radiotherapy in prostate cancer: first results from the MRC RT01 randomised controlled trial	Article	2007	David P Dearnaley	The Lancet. Oncology	482
10	Phase III postoperative adjuvant radiotherapy after radical prostatectomy compared with radical prostatectomy alone in pT3 prostate cancer with postoperative undetectable prostate-specific antigen: ARO 96-02/AUO AP 09/95	Article	2009	Thomas Wiegel	Journal of clinical oncology : official journal of the American Society of Clinical Oncology	480

**Figure 8:**
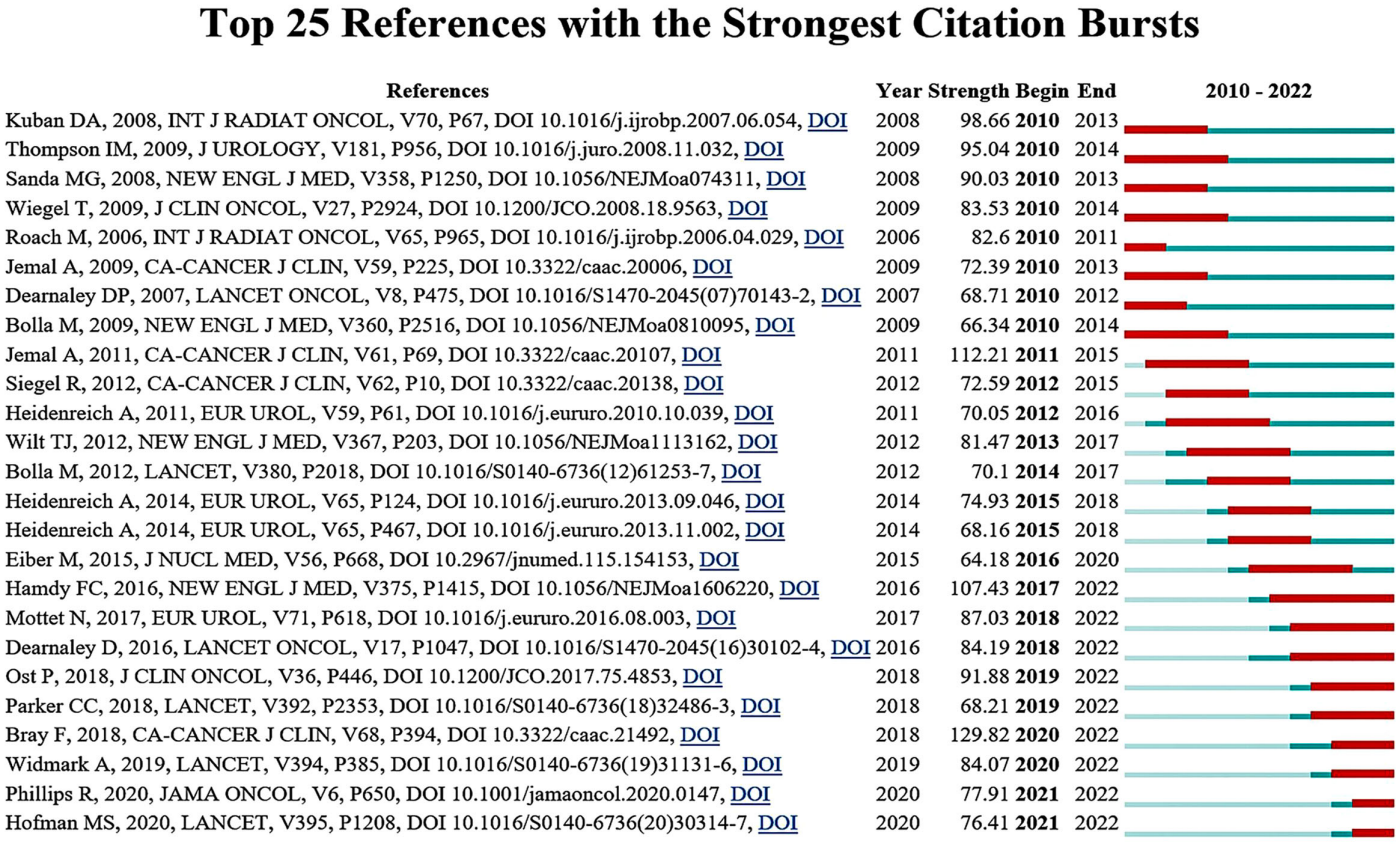
Top 25 references with the strongest citation bursts.

Furthermore, the burstness analysis provides insights into the development of research hotspots and trends over a period. We performed a burstness analysis of the references, and the top 25 are listed in **Figure 8**. Bray F, 2018, CA-CANCER J CLIN, V68, P394 had the highest burst strength (n=129.82), entitled *Global cancer statistics 2018: GLOBOCAN estimates of incidence and mortality worldwide for 36 cancers in 185 countries*, with citation burstness from 2020 to 2022. Notably, two references are still frequently cited in the last two years. Respectively, Ryan Phillips et al. determined that stereotactic ablative radiotherapy (SABR) could improve oncological outcomes in patients with oligometastatic prostate cancer (MPC); Michael S Hofman et al. highlighted that prostate-specific membrane antigen (PSMA) PET-CT could provide a more accurate and effective basis for the management for PCa patients prior to RT or for the detection of BCR after radical RT.

### 3.5 Analysis of keywords

#### 3.5.1 Co-occurrence analysis of keywords

**Figure 9:**
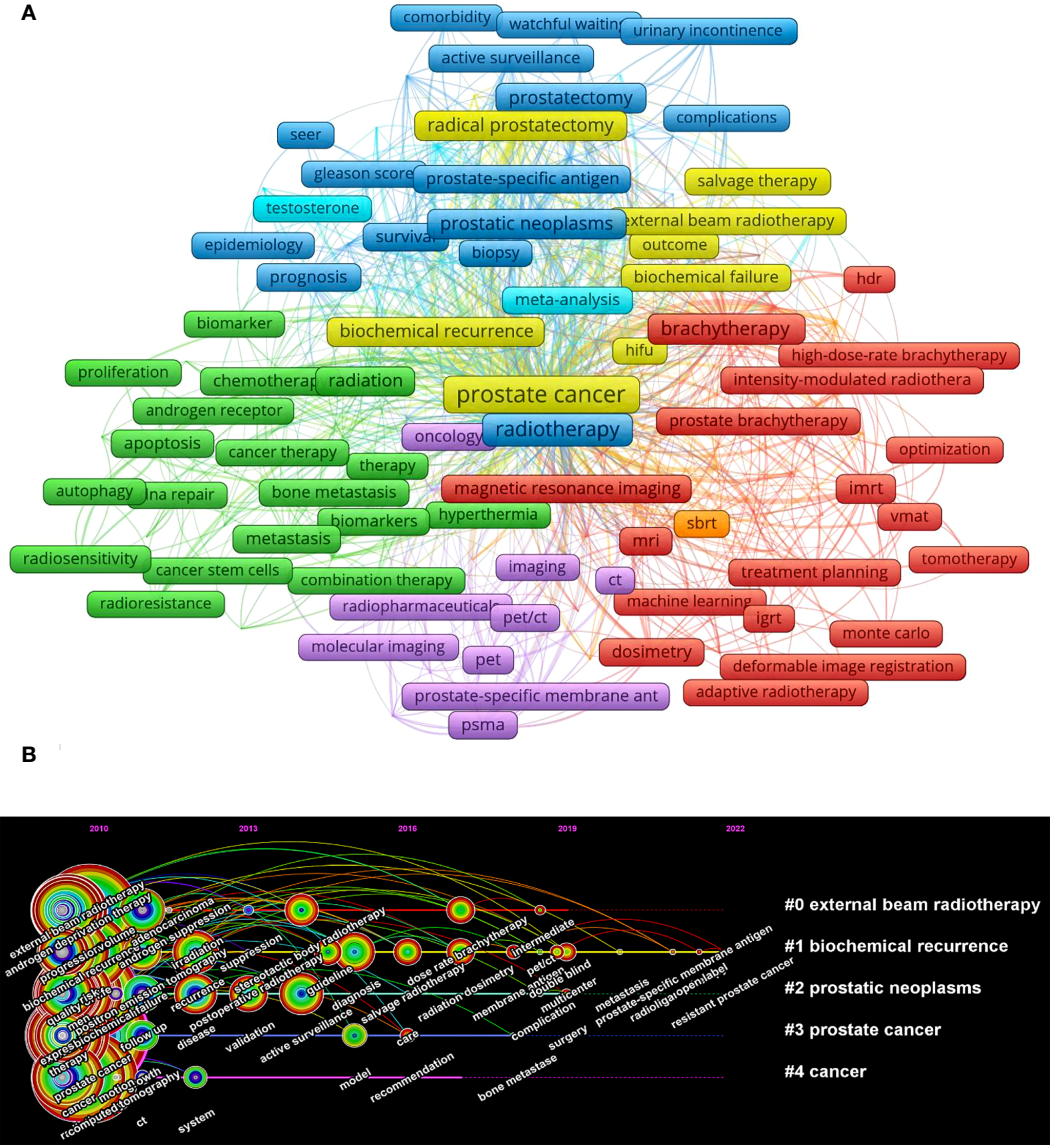
Visualization map of keywords in publications. **(A)** Occurrence analysis of keywords. **(B)** Timeline view of keywords. Each horizontal line indicates a cluster. The size of the circle indicates the frequency of occurrence, with the color approaching red representing the closer time.

Among the 24267 keywords, 165 appeared at least 50 times (**Figure 9A**). **Table 5** shows the top 10 keywords about frequency and centrality. The most frequent term was “prostate cancer” (n=6755), followed by “radiotherapy” (n=2165) and “brachytherapy” (n=1156). According to the centrality, the term with the highest centrality was “prostate cancer” (n=0.81), followed by “radical prostatectomy” (n=0.4) and “radiation therapy” (n=0.15). RT and RP exhibit distinct advantages in the comprehensive management of prostate cancer, and the comparative analysis of the two modalities has consistently remained a focal point of interest for researchers. A research report on a fifteen-year follow-up study of localized prostate cancer was recently published in *THE NEW ENGLAND JOURNAL OF MEDICINE* on April 27, 2023. The study findings reveal that both RP and RT demonstrate notably low prostate cancer-specific mortality (14). In addition, the timeline view of keywords shows the high-frequency keywords in each cluster over time (**Figure 9B**). The cluster #1(biochemical recurrence) is still ongoing, which provides researchers with a reference for research hotspots.

**Table 5:** Top 10 keywords according to the frequency and centrality.

Rank	Keywords	Counts	Rank	Keywords	Centrality
1	Prostate cancer	6755	1	Prostate cancer	0.81
2	Radiotherapy	2165	2	Radical prostatectomy	0.4
3	Brachytherapy	1156	3	Radiation therapy	0.15
4	Prostatic neoplasms	735	4	Diagnosis	0.15
5	Radiation therapy	707	5	Progression	0.13
6	Radical prostatectomy	653	6	Dose escalation	0.13
7	Prostatectomy	559	7	Cancer	0.12
8	Quality of life	473	8	Androgen deprivation therapy	0.08
9	Radiation	389	9	Multicenter	0.08
10	Biochemical recurrence	355	10	Biochemical recurrence	0.07

#### 3.5.2 Burst keyword analysis

**Figure 10:**
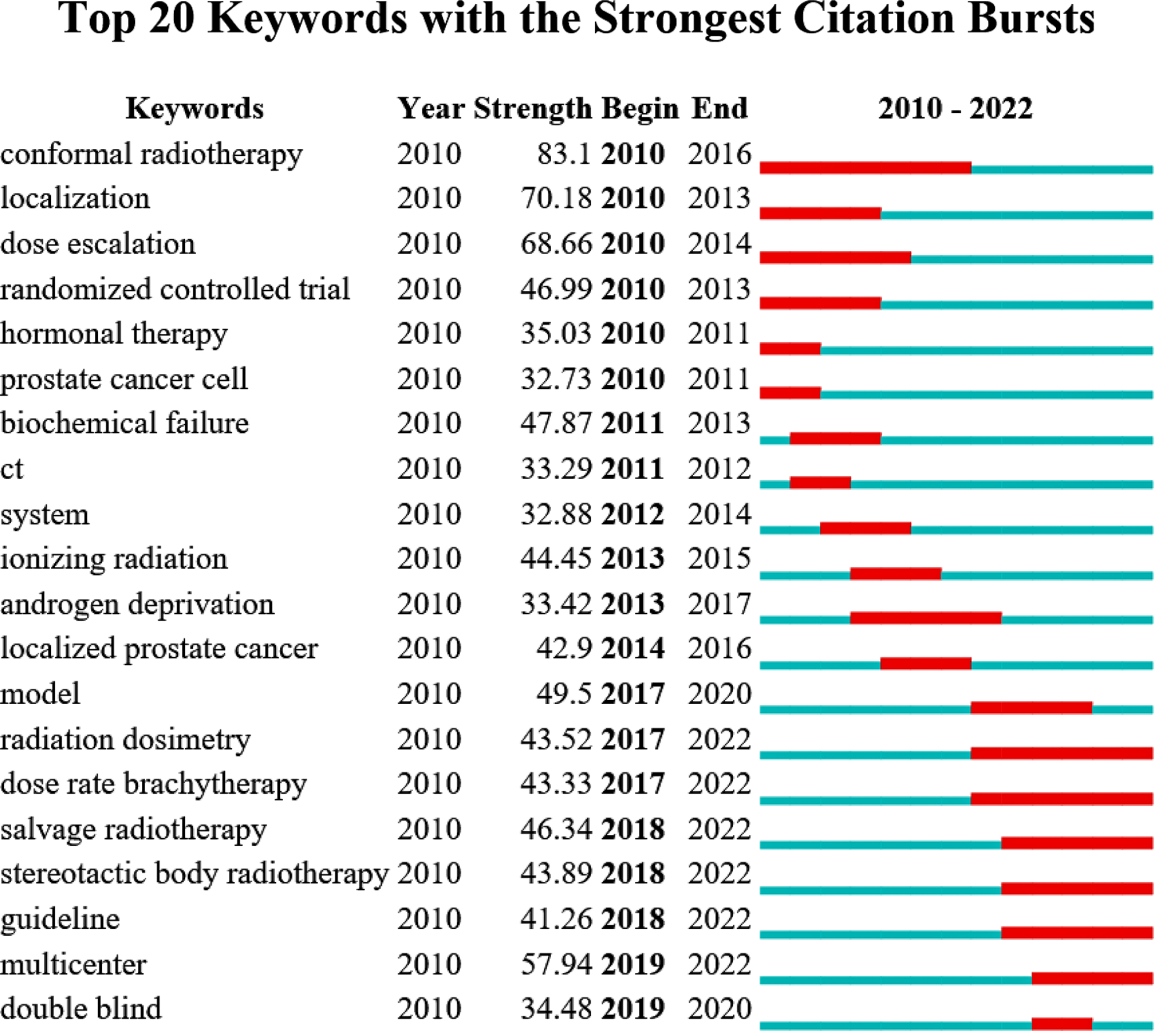
Top 25 keywords with the strongest citation bursts.

The bursts analysis is based on the word frequency growth to screen out words with high-frequency change rates and fast growth rates. As shown in **Figure 10**, the term with the strongest burst strength was “conformal radiotherapy”(n=83.1), followed by “localization” (n=70.18) and “dose escalation” (n=68.66). Apparently, the word with the good burst strength in the past 2 years was “radiation dosimetry”, “dose rate brachytherapy”, “salvage radiotherapy”, “stereotactic body radiotherapy”, “guideline”, and “multicenter”.

## 4 Discussion

### 4.1 General information

In terms of the global publication volume over the last decade, there has been a general upward trend in research related to RT for PCa. Analysis of countries/regions shows that USA ranks first in the world in terms of the number of publications and citations. The majority (90%) of the top ten institutions are affiliated with the USA, further demonstrating the dominance of the USA in this field. This is due to the long-term advanced level in the medical field of European and American countries led by the USA. Among the university institutions, the University of Toronto, which is affiliated to Canada, ranks first in the number of articles published, and its contribution in the field also deserves our close attention. It is worth noting that while China holds the second position in terms of total publications, total citations for its research do not attain a leading position. This disparity implies that there exists potential for enhancing the innovativeness, breadth, or depth of China’s relevant research endeavors. Chinese researchers should strengthen their collaboration and conduct more high-quality and innovative basic or clinical trials to increase China’s academic influence. Furthermore, cooperation between countries/regions is mainly concentrated in countries such as the USA, UK, Netherlands, and China. Global cooperation requires to be further strengthened.

Among the top 10 authors according to publications and co-citations, A V D’Amico ranks among the top 5. A V D’Amico has made great contributions to the field of RT on PCa. In 1998, his team published an article, reporting that patients with intermediate and high-risk PCa who underwent RP or external beam radiation (EBRT) showed better BCR outcomes than those who received interstitial radiation therapy, which has been co-cited up to 1182 times in the field. Additionally, Paul L Nguyen, who has published the largest number of articles (n=179), has also made outstanding contributions to the research of RT on PCa. In 2018, Paul L Nguyen et al. published an article in the journal *CANCER* entitled *Travel distance and stereotactic body radiotherapy for localized prostate cancer*. The article emphasized the growing interest in the therapeutic effect and significance of definitive stereotactic body radiotherapy (SBRT) in localized PCa (15). Notably, the most co-cited author is Michael J Zelefsky (n=3376). Just in September 2022, Michael J Zelefsky et al. published an article entitled *Combined brachytherapy and ultra-hypofractionated radiotherapy for intermediate-risk prostate cancer: Comparison of toxicity outcomes using a high-dose-rate (HDR) versus low-dose-rate (LDR) brachytherapy boost*. They reported that both LDR and HDR brachytherapy boost combined with ultra-hypofractionated external beam radiation therapy (UH-EBRT) had good toxicity profiles, with a significant reduction in grade 2+ genitourinary toxicity found in patients receiving HDR (16).

Our journal analysis shows that the related journals are mainly concerned with clinical medicine, molecular biology, and immunology, which is consistent with the dual-map analysis. The journal *INTERNATIONAL JOURNAL OF RADIATION ONCOLOGY BIOLOGY PHYSICS* published the largest number of papers(n=1026) in the field, also ranking first in terms of co-citations (n=78550). This journal has received widespread attention from researchers. The journals *RADIOTHERAPY AND ONCOLOGY* and *MEDICAL PHYSICS* have an important influence on research, ranking second and third respectively in the number of publications. It is worth noting that although the journal *JAMA* is not in the top 10 in terms of publications and co-citations, three of the top 10 most co-cited articles were published in this journal, accounting for the largest proportion (30%), which deserves attention of the researchers. Important outputs in the field may later be published in the above journals. Researchers can regard these journals as an important source of theoretical references and ideal choices for publication in future.

The collation of high-frequency co-cited references provides an understanding about the knowledge base in the field. Among the top 10 co-cited references, seven mainly focus on the impact of RT on the outcome indicators such as BCR in PCa patients, and four explored the effectiveness of RT at different doses for PCa patients. Burstness analysis of references showed two references in burst which deserve our attention because they highlight the important role of PSMA PET-CT in guiding RT strategies.

### 4.2 Hotspots and trends of radiotherapy on prostate cancer

Amid the ongoing information explosion, it is vital for researchers to effectively grasp the developmental trends in their research field. In this paper, we utilized bibliometrics to explore emerging topics in the field through the cluster analysis and citation burstness analysis of references (5, 9, 17). Then, we evaluated the hotspots and frontiers through keyword co-occurrence analysis (18), keyword timeline (19), and burstness analysis of keywords.

Cluster analysis of references showed that RT for OMPC has been a hot spot in recent years. OMPC is a type of PCa between the state of tumor localization and extensive metastasis (20). The 2021 Updated European Association of Urology guidelines recommend a regimen of androgen deprivation therapy (ADT) combined with RT for patients with OMPC (21). SBRT, as a non-invasive treatment, can provide good control of local tumors with shorter treatment cycles and larger single doses (22). SBRT is a breakthrough treatment in the field of RT and has received widespread attention, which is in line with the results of our keyword burstness analysis. Some scholars evaluated 117 lesions in 74 patients with pelvic node oligorecurrent PCa who were treated with SBRT, and the result showed a 100% local control rate in all patients (23). In a prospective study, Deodato et al. selected 37 OMPC patients with bone metastases who received single fraction SBRT in the dose range of 12-24 Gy. During the median follow-up period of 25 months, few toxic events were observed in these patients, showing a high local control rate and prolonged *next-line* *systemic* *treatment-free* survival (NEST-FS) (24). In addition, the reference burstness analysis showed that (PSMA) PET-CT has gradually been widely used in recent years. Mazzola et al. conducted a prospective observational study involving 20 patients with castration sensitive oligorecurrent PCa who underwent PSMA-PET/CT guided SBRT by means of 1.5 T MRI-Linac, which initially confirmed the effectiveness and tolerability of this treatment (25). Other studies have shown that compared with choline-PET, PSMA-11-PET-guided SBRT resulted in a significantly longer response duration and ADT-free survival (26). A multi-institutional study in 2022 also demonstrated the superior performance of PSMA-PET guided SBRT in delaying the initiation of ADT in OMPC patients (27). PSMA-PET imaging holds great promise in the treatment of PCa. Nevertheless, the biological characteristics of OMPC are not fully understood, and there is no international consensus on the management of OMPC. The inclusion of SBRT in the routine management of PCa currently requires long-term clinical studies.

Our keyword burstness analysis showed that brachytherapy (BT) has also been a focus of research in the field of RT for PCa in the past 5 years. BT mainly consists of low-dose rate brachytherapy (LDR-BT) and high-dose rate brachytherapy (HDR-BT). Although HDR-BT requires higher equipment costs than LDR-BT, several studies in the last two years have shown that HDR-BT is significantly better than LDR-BT in terms of postoperative adverse effects. Parry et al. conducted an observational cohort study of 54642 PCa patients and showed that both HDR-BT and LDR-BT exhibited similar degrees of genitourinary (GU) toxicity, whereas LDR-BT had significantly worse gastrointestinal (GI) toxicity (28). By enrolling 99 patients with intermediate-risk PCa, Kollmeier et al. demonstrated that patients receiving HDR-BT exhibited significantly less grade 2+ GU toxicity than those receiving LDR-BT (16). Other studies indicated that HDR-BT has better health related quality of life (HRQOL) in the irritative urinary domain compared with LDR-BT, although LDR-BT resulted in lower nadir prostate-specific antigen (nPSA) (29, 30). In addition, as an important modality for salvage RT, HDR-BT has similar efficacy to LDR-BT (31), while HDR-BT has potential advantages due to its biological characteristics and uneven dose distribution. Ménard et al. studied 88 patients from two institutions who underwent salvage HDR-BT at 22-26 Gy, and the 3-year and 5-year failure-free survival (FFS) rates were 67% and 49%, respectively (32). Kissel et al. reported 64 patients treated with salvage HDR-BT and showed a 2-year disease-free survival (DFS) rate of 58% in the whole population and 66% in hormone-sensitive patients (33). Given the lack of data from large-scale phase III clinical trials and no consensus on the optimal fractionation schedule, the potential of HDR-BT in the treatment of PCa needs to be further explored at a later stage.

Through the analysis of the timeline of keywords, it is apparent to see that the current research focus in the field of RT for PCa continues to revolve around the BCR after RT. BCR after RT is defined as a PSA value above the nadir of 2ng/ml after RT (34). The BCR has guided researchers to explore protocol options and treatment outcomes for multiple RT modalities, and has also prompted researchers to introduce more sensitive and accurate detection devices (such as PSMA-PET), which significantly enhanced the ability to localize PCa recurrence. These are inseparable from the implementation of many multicenter studies in the past two years (35–39), which is similar to the results of our keyword burstness analysis. In the field of RT for PCa, more multicenter clinical trials may emerge in the next few years, giving researchers new insights.

### 4.3 Strength and limitations

Compared with the previous meta-analyses and reviews, this bibliometric analysis provides more important data about the characteristics of RT for PCa, more objective references for the developmental trends and hotspots in the field, and a clearer picture of RT for PCa from multiple dimensions. Furthermore, different from previous investigations (6), this manuscript presents an immensely comprehensive and state-of-the-art data compilation and places particular emphasis on comprehensively exploring the panorama and advancements of RT in PCa from the entire spectrum of the field, aiming to contribute a wealth of content to the current knowledge system from a macro perspective. In data analysis, this paper has employed not only CiteSpace but also VOSviewer, another widely utilized tool in the field of bibliometrics. The latter furnishes an extensive array of visualization options, encompassing network visualization, density visualization, and overlay visualization, thereby empowering researchers to explore and present bibliometric data from diverse formats and perspectives. The synergistic amalgamation of these two tools enhances the visualization efficacy, credibility, and robustness of our research outcomes. In research hotspots, this study includes an essential analysis of keywords, including co-occurrence analysis, timeline, and burstness analysis. These analytical approaches, which have not been previously explored, provide novel insights into the underlying patterns and dynamics within the research domain. Novel hotspots and frontiers have been discerned, revealing the current research emphasis in RT for PCa revolving around the issue of BCR. Moreover, over the past two years, BT and SBRT have emerged as the central themes within this domain. Of the two, at least BT was overlooked in the previous study.

Certainly, there are inevitably some limitations in this study. This study only included original articles and reviews in English from the WoSCC database, which may differ slightly from the actual results. In addition, the constant updating of the database also had a subtle impact on the results of analysis, and more research needs to be included for future refinement.

## 5 Conclusion

Research on RT for PCa has been growing gradually worldwide over the last decade, with an emphasis on OMPC currently. The continuous advancement of imaging technologies has unveiled significant prospects for SBRT and BT in the realm of PCa treatment. Moreover, addressing the issue of BCR in PCa has long been a matter of importance. In this regard, salvage radiotherapy has garnered significant attention as a closely associated area of investigation at present. Several related large-scale multicenter studies have been conducted in recent years. More high-quality research is expected to be employed to guide clinical decision-making.

## Data availability statement

The raw data supporting the conclusions of this article will be made available by the authors, without undue reservation.

## Author contributions

Study concept and design: PHL, XX, PZ, and CR. Acquisition of data: XX and PZ. Analysis and interpretation of data: XX, PZ, CR, LL, JH, and PL. Drafting of the manuscript: XX, PZ, and CR. Critical revision of the manuscript for important intellectual content: XX and PHL. Statisticalanalysis: XX, PZ, CR, LL, JH, and PL. Supervision: PHL. All authors contributed to the article and approved the submitted version.

## References

[1] Bray F Ferlay J Soerjomataram I Siegel RL Torre LA Jemal A. Global cancer statistics 2018: GLOBOCAN estimates of incidence and mortality worldwide for 36 cancers in 185 countries. CA: Cancer J For Clin (2018) 68(6):394–424. doi: 10.3322/caac.21492 30207593

[2] Lehrer EJ Singh R Wang M Chinchilli VM Trifiletti DM Ost P. Safety and survival rates associated with ablative stereotactic radiotherapy for patients with oligometastatic cancer: A systematic review and meta-analysis. JAMA Oncol (2021) 7(1):92–106. doi: 10.1001/jamaoncol.2020.6146 PMC7689573 33237270

[3] Spratt DE Malone S Roy S Grimes S Eapen L Morgan SC. Prostate radiotherapy with adjuvant androgen deprivation therapy (ADT) improves metastasis-free survival compared to neoadjuvant ADT: an individual patient meta-analysis. J Clin Oncol Off J Am Soc Clin Oncol (2021) 39(2):136–44. doi: 10.1200/JCO.20.02438 PMC8189640 33275486

[4] Yeung AWK Tosevska A Klager E Eibensteiner F Laxar D Stoyanov J. Virtual and augmented reality applications in medicine: analysis of the scientific literature. J Med Internet Res (2021) 23(2):e25499. doi: 10.2196/25499 33565986 PMC7904394

[5] Chen C Song M. Visualizing a field of research: A methodology of systematic scientometric reviews. PloS One (2019) 14(10):e0223994. doi: 10.1371/journal.pone.0223994 31671124 PMC6822756

[6] Li R Liu X Yang B Qiu J. External beam radiotherapy for prostate cancer: What are the current research trends and hotspots? Cancer Med (2021) 10(2):772–82. doi: 10.1002/cam4.3700 PMC7877352 33480190

[7] Chen C. Searching for intellectual turning points: progressive knowledge domain visualization. Proc Natl Acad Sci United States America (2004) 101 Suppl 1:5303–10. doi: 10.1073/pnas.0307513100 PMC387312 14724295

[8] Chen C. CiteSpace II: Detecting and visualizing emerging trends and transient patterns in scientific literature. J Am Soc Inform Sci Technol (2006) 57(3):359–77. doi: 10.1002/asi.20317

[9] Chen C. Science mapping: A systematic review of the literature. J Data Inf Sci (2017) 2(2):1–40. doi: 10.1515/jdis-2017-0006

[10] van Eck NJ Waltman L. Software survey: VOSviewer, a computer program for bibliometric mapping. Scientometrics (2010) 84(2):523–38. doi: 10.1007/s11192-009-0146-3 PMC2883932 20585380

[11] Rad AE Brinjikji W Cloft HJ Kallmes DF. The H-index in academic radiology. Acad Radiol (2010) 17(7):817–21. doi: 10.1016/j.acra.2010.03.011 20471868

[12] Rozet F Mongiat-Artus P Hennequin C Beauval JB Beuzeboc P Cormier L. [French ccAFU guidelines - update 2020-2022: prostate cancer]. Progres en Urologie J L'Association Francaise D'urologie Et la Societe Francaise D'urologie (2020) 30(12S):S136–251. doi: 10.1016/S1166-7087(20)30752-1 33349424

[13] Chang Y Zhao X Xiao Y Yan S Xu W Wang Y. Neoadjuvant radiohormonal therapy for oligo-metastatic prostate cancer: safety and efficacy outcomes from an open-label, dose-escalation, single-center, phase I/II clinical trial. Front Med (2023) 17(2):231–9. doi: 10.1007/s11684-022-0939-9 36580231

[14] Hamdy FC Donovan JL Lane JA Metcalfe C Davis M Turner EL. Fifteen-year outcomes after monitoring, surgery, or radiotherapy for prostate cancer. New Engl J Med (2023) 388(17):1547–58. doi: 10.1056/NEJMoa2214122 36912538

[15] Mahal BA Chen Y-W Sethi RV Padilla OA Yang DD Chavez J. Travel distance and stereotactic body radiotherapy for localized prostate cancer. Cancer (2018) 124(6):1141–9. doi: 10.1002/cncr.31190 29231964

[16] Kollmeier MA Gorovets D Flynn J McBride S Brennan V Beaudry J. Combined brachytherapy and ultra-hypofractionated radiotherapy for intermediate-risk prostate cancer: Comparison of toxicity outcomes using a high-dose-rate (HDR) versus low-dose-rate (LDR) brachytherapy boost. Brachytherapy (2022) 21(5):599–604. doi: 10.1016/j.brachy.2022.04.006 35725549 PMC10372465

[17] Ma L Ma J Teng M Li Y. Visual analysis of colorectal cancer immunotherapy: A bibliometric analysis from 2012 to 2021. Front In Immunol (2022) 13:843106. doi: 10.3389/fimmu.2022.843106 35432385 PMC9009266

[18] Xiao F Li C Sun J Zhang L. Knowledge domain and emerging trends in organic photovoltaic technology: A scientometric review based on citeSpace analysis. Front In Chem (2017) 5:67. doi: 10.3389/fchem.2017.00067 28966923 PMC5605557

[19] Wang Y Jia Y Li M Jiao S Zhao H. Hotspot and frontier analysis of exercise training therapy for heart failure complicated with depression based on web of science database and big data analysis. Front In Cardiovasc Med (2021) 8:665993. doi: 10.3389/fcvm.2021.665993 34095256 PMC8169975

[20] Rossetti S Di Napoli M Pisano C Cecere SC Tambaro R Ventriglia J. Oligometastatic prostate cancer treatment. Future Oncol (London England) (2021) 17(29):3893–9. doi: 10.2217/fon-2021-0126 34296622

[21] Cornford P van den Bergh RCN Briers E Broeck den Van T Cumberbatch MG Santis De M. EAU-EANM-ESTRO-ESUR-SIOG guidelines on prostate cancer. Part II-2020 update: treatment of relapsing and metastatic prostate cancer. Eur Urol (2021) 79(2):263–82. doi: 10.1016/j.eururo.2020.09.046 33039206

[22] Ghadjar P Wiegel T De Bleser E Jereczek-Fossa BA Pasquier D. Metastasis-directed therapy in treating nodal oligorecurrent prostate cancer: A multi-institutional analysis comparing the outcome and toxicity of stereotactic body radiotherapy and elective nodal radiotherapy. Eur Urol (2019) 76:732–9. doi: 10.1016/j.eururo.2019.07.009 31331782

[23] Cozzi S Botti A Timon G Blandino G Najafi M Manicone M. Prognostic factors, efficacy, and toxicity of involved-node stereotactic body radiation therapy for lymph node oligorecurrent prostate cancer : An investigation of 117 pelvic lymph nodes. Strahlentherapie Und Onkologie Organ Der Deutschen Rontgengesellschaft (2022) 198(8):700–9. doi: 10.1007/s00066-021-01871-5 34757443

[24] Deodato F Pezzulla D Cilla S Ferro M Romano C Bonome P. Stereotactic radiosurgery for bone metastases in oligometastatic prostate cancer patients: DESTROY-2 clinical trial subanalysis. Clin Trans Oncol Off Publ Fed Spanish Oncol Societies Natl Cancer Institute Mexico (2022) 24(6):1177–83. doi: 10.1007/s12094-021-02764-w 34984604

[25] Mazzola R Cuccia F Figlia V Rigo M Nicosia L Giaj-Levra N. Stereotactic body radiotherapy for oligometastatic castration sensitive prostate cancer using 1.5 T MRI-Linac: preliminary data on feasibility and acute patient-reported outcomes. La Radiologia Med (2021) 126(7):989–97. doi: 10.1007/s11547-021-01352-w 33835309

[26] Deijen CL Vrijenhoek GL Schaake EE Vogel WV Moonen LMF Pos FJ. PSMA-11-PET/CT versus choline-PET/CT to guide stereotactic ablative radiotherapy for androgen deprivation therapy deferral in patients with oligometastatic prostate cancer. Clin Trans Radiat Oncol (2021) 30:1–6. doi: 10.1016/j.ctro.2021.06.004 PMC8261473 34278008

[27] Mazzola R Cuccia F Pastorello E Salgarello M Francolini G Livi L. PSMA-guided metastases directed therapy for bone castration sensitive oligometastatic prostate cancer: a multi-institutional study. Clin Exp Metastasis (2022) 39(3):443–8. doi: 10.1007/s10585-022-10157-8 35266063

[28] Parry MG Nossiter J SujenthIran A Cowling TE Patel RN Morris M. Impact of high-dose-rate and low-dose-rate brachytherapy boost on toxicity, functional and cancer outcomes in patients receiving external beam radiation therapy for prostate cancer: A national population-based study. Int J Radiat Oncol Biol Phys (2021) 109(5):1219–29. doi: 10.1016/j.ijrobp.2020.11.023 33279595

[29] Levin-Epstein R Cook RR Wong JK Stock RG Demanes Jeffrey D Collins SP. Prostate-specific antigen kinetics and biochemical control following stereotactic body radiation therapy, high dose rate brachytherapy, and low dose rate brachytherapy: A multi-institutional analysis of 3502 patients. Radiother Oncol J Eur Soc For Ther Radiol Oncol (2020) 151:26–32. doi: 10.1016/j.radonc.2020.07.014 32663537

[30] Reynaud T Hathout L Carignan D Barkati M Martin A-G Foster W. PSA outcomes and late toxicity of single-fraction HDR brachytherapy and LDR brachytherapy as monotherapy in localized prostate cancer: A phase 2 randomized pilot study. Brachytherapy (2021) 20(6):1090–8. doi: 10.1016/j.brachy.2021.05.010 34238688

[31] Kollmeier MA McBride S Taggar A Anderson E Lin M Pei X. Salvage brachytherapy for recurrent prostate cancer after definitive radiation therapy: A comparison of low-dose-rate and high-dose-rate brachytherapy and the importance of prostate-specific antigen doubling time. Brachytherapy (2017) 16(6):1091–8. doi: 10.1016/j.brachy.2017.07.013 28838648

[32] Ménard C Navarro-Domenech I Liu ZA Joseph L Barkati M Berlin A. MRI-guided focal or integrated boost high dose rate brachytherapy for recurrent prostate cancer. Front In Oncol (2022) 12:971344. doi: 10.3389/fonc.2022.971344 PMC9459480 36091157

[33] Kissel M Pounou A Ka K Alexis A Irani J Jereczek-Fossa BA. Efficacy and toxicity following salvage high-dose-rate brachytherapy for locally recurrent prostate cancer after radiotherapy. Brachytherapy (2022) 21(4):424–34. doi: 10.1016/j.brachy.2022.01.005 35331666

[34] Cornford P Bellmunt J Bolla M Briers E De Santis M Gross T. EAU-ESTRO-SIOG guidelines on prostate cancer. Part II: treatment of relapsing, metastatic, and castration-resistant prostate cancer. Eur Urol (2017) 71(4):630–42. doi: 10.1016/j.eururo.2016.08.002 27591931

[35] Cerci JJ Fanti S Lobato EE Kunikowska J Alonso O Medina S. Diagnostic performance and clinical impact of ga-PSMA-11 PET/CT imaging in early relapsed prostate cancer after radical therapy: A prospective multicenter study (IAEA-PSMA study). J Nucl Med Off Publication Soc Nucl Med (2022) 63(2):240–7. doi: 10.2967/jnumed.120.261886 PMC8805782 34215674

[36] Kirste S Kroeze SGC Henkenberens C Schmidt-Hegemann N-S Vogel MME Becker J. Combining ga-PSMA-PET/CT-directed and elective radiation therapy improves outcome in oligorecurrent prostate cancer: A retrospective multicenter study. Front In Oncol (2021) 11:640467. doi: 10.3389/fonc.2021.640467 PMC8141738 34041020

[37] Kishan AU Wang X Sun Y Romero T Michalski JM Ma TM. High-dose radiotherapy or androgen deprivation therapy (HEAT) as treatment intensification for localized prostate cancer: an individual patient-data network meta-analysis from the MARCAP consortium. Eur Urol (2022) 82(1):106–14. doi: 10.1016/j.eururo.2022.04.003 35469702

[38] Kroeze SGC Henkenberens C Schmidt-Hegemann NS Vogel MME Kirste S Becker J. Prostate-specific membrane antigen positron emission tomography-detected oligorecurrent prostate cancer treated with metastases-directed radiotherapy: role of addition and duration of androgen deprivation. Eur Urol Focus (2021) 7(2):309–16. doi: 10.1016/j.euf.2019.08.012 31495759

[39] Spohn SKB Farolfi A Schandeler S Vogel MME Ruf J Mix M. The maximum standardized uptake value in patients with recurrent or persistent prostate cancer after radical prostatectomy and PSMA-PET-guided salvage radiotherapy-a multicenter retrospective analysis. Eur J Nucl Med Mol Imaging (2022) 50(1):218–27. doi: 10.1007/s00259-022-05931-5 PMC9668780 35984452

